# Spontaneous Urinoma Without Trauma or Obstruction in a 64-Year-Old Female

**DOI:** 10.7759/cureus.9241

**Published:** 2020-07-17

**Authors:** Patrick S Finnegan, Thomas Proctor, Brian Pennington

**Affiliations:** 1 Emergency Medicine, Grandview Medical Center - Kettering Health Network, Dayton, USA; 2 Emergency Medicine, Kettering Health Network, Dayton, USA

**Keywords:** urinoma, emergency medicine, case report

## Abstract

A 64-year-old female presented to the ED with severe abdominal pain. Initially it was suspected to be spontaneous aortic rupture or dissection. Contrasted CT imaging studies did not identify aneurysm and dissection, but did identify a concerning, confounding, and curious collection of fluid in the upper right quadrant. Angiography imaging was obtained and identified the origin as the collecting duct of the right kidney. The patient was admitted to the medical service. She was then evaluated by the urology service and they identified this presentation as a urinoma with extravasation of urine, in the absence of trauma or identifiable obstruction.

## Introduction

The spontaneous rupture of the ureter is a rare presentation to the ED. Generally, there are three common origins for spontaneous ureter rupture. The first is iatrogenic through surgical procedure usually open abdominal surgeries. The second is traumatic, most commonly puncture injuries than blast injuries. And the third is obstructive process. This would be a ureterolithiasis that disrupts urine movement internally or a compressive mass on the ureter that inhibits urine movement through compression. This is a case report for a patient with no known recent surgical interventions, traumatic injuries, or intrabdominal processes which appears to make this case novel and unique in emergency medicine medical literature [1-2].

## Case presentation

A 64-year-old female presented to the ED complaining of sudden, severe, unrelenting right upper quadrant/epigastric abdominal pain that radiated to her back. She stated that it started while she was playing card games with family members. She denied any recent traumatic event. She had associated emesis that was nonbloody and nonbilious. She denied any fevers, chills, urinary symptoms such as frequency, urgency, hematuria, dysuria, or discharge. Her past medical history included type 2 diabetes mellitus, diverticulitis, GERD, and arthritis. She had no recent surgical interventions but had had a prior tonsillectomy, hysterectomy, and knee arthroscopy the most recent procedure in 2013.

In the ED, the primary concerning differential based upon presentation was aortic dissection versus aneurysm. Pertinent laboratory abnormalities included a serum creatinine of 1.4, an increase from her previous baseline of 0.9 from prior visits; serum glucose of 348, consistent with poorly controlled diabetes; elevated lactic acid of 4.2 with a repeat value of 1.7 following fluid administration. The patient had moderate blood in urine and glucose greater than 1000 mg/dL, but the blood was not hematuric. The patient had unremarkable liver function tests, serum lipase, and venous blood gas. The patient did not have a leukocytosis.

**Figure 1:**
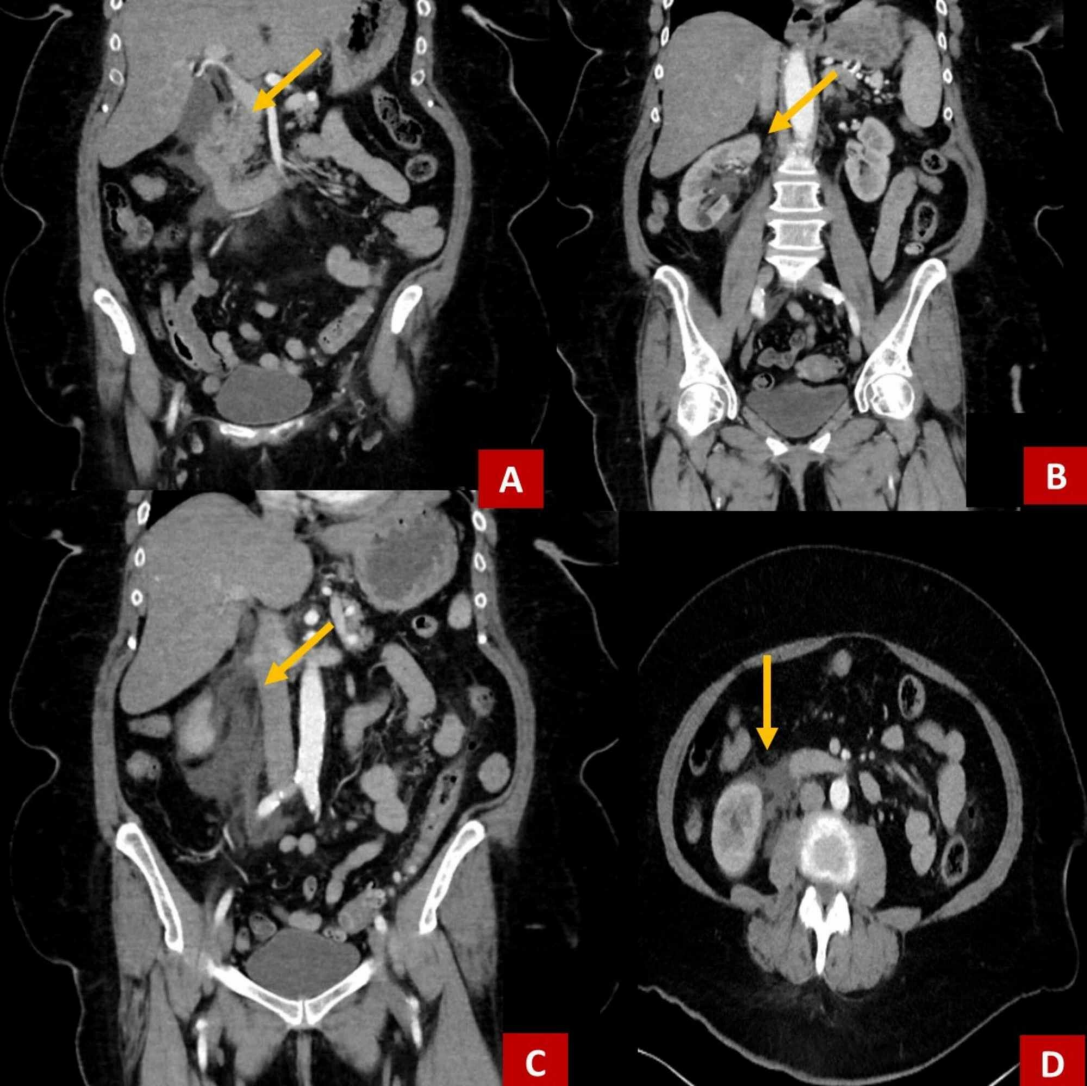
CT abdomen and pelvis coronal and transverse planes. Demonstrates fluid in the upper right quadrant near the kidney, stomach, and duodenum. Initial imaging was unclear of the etiology for this fluid. A,B,C: coronal plane, D: transverse plane.

**Figure 2:**
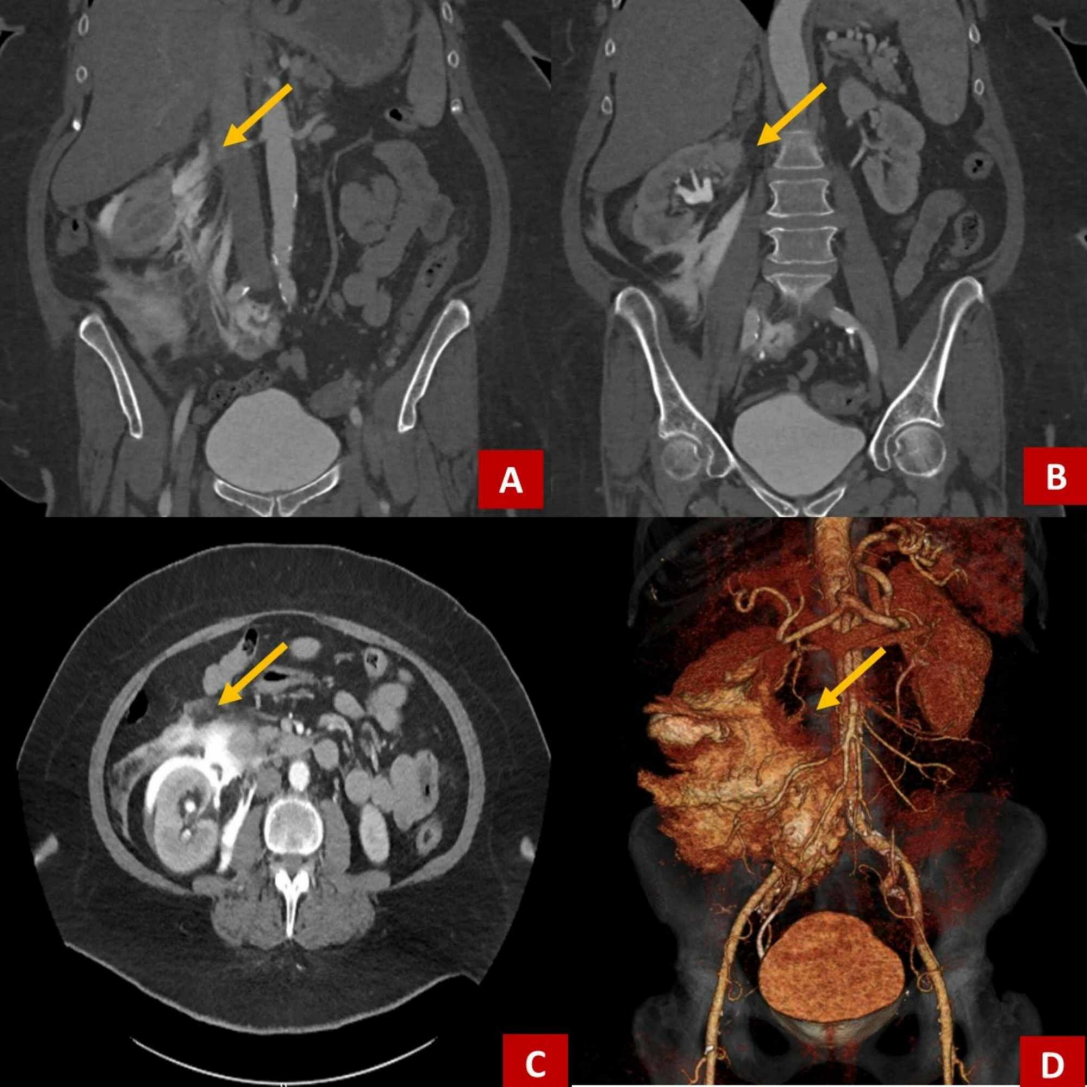
CT abdomen and pelvis with angiography coronal and transverse planes. Demonstrates fluid in the upper right quadrant near the kidney, stomach, and duodenum. This imaging suggested renal or urethral origin of the fluid suspected to be a urinoma at the time of disposition. A,B: coronal plane, C: transverse plane, D: 3D reconstruction.

A CT scan of the abdomen and pelvis was obtained with contrasted dye. It identified hydronephrosis and hydroureter without and identifiable obstruction around the right kidney (Figure 1). The source was thought to be nephrotic; however, the CT imaging was unable to rule out intra-abdominal bleeding from the stomach or duodenum. There was no evidence of aortic aneurysm or dissection. To better assess the presenting process, CT angiography of the abdomen and pelvis was performed. This identified the presence of contrast extravasation suggestive of a right-sided urinoma (Figure 2).

 

During the ED course, her pain was controlled with morphine, her nausea was controlled with promethazine and ondansetron, and a bolus liter of normal saline for the elevated lactic acid. The patient was admitted to the hospitalist service and the urology service was consulted.

The patient was taking to the operating suite the following day. The urology team performed a retrograde pyelogram and placed a double J stent in the ureter. The patient tolerated the procedure well and repeat imaging of the abdomen identified interval improvement of the fluid collection and no evidence of extravasation.

## Discussion

The most common cause of ureteral injury is iatrogenic surgical trauma. The injury is usually identified during the procedure and is repaired prior to significant urinoma development. Following this, less than 4% of penetrating traumatic insults and of 1% blunt traumatic insults resulted in ureteral injuries [1-6]. Because of the infrequency in traumatic presentation and the general origin being surgical intervention, it is a rare presentation in the ED.

Therefore, ED presentation of spontaneous urinoma is extremely rare in the literature. A review of PubMed studies suggests that there are three general origins for a urinoma: an obstructive process in addition to the already mentioned traumatic incidents or iatrogenic origins. Obstructive processes can include ureterolithiasis, retroperitoneal fibrosis, or intra-abdominal mass effect [3-4].

For this patient, an iatrogenic cause is unlikely with no recent abdominal surgeries. The patient had a hysterectomy in the past, but it was over 10 years prior to presentation to the ED. The patient denied traumatic incident. There were no penetrating injuries upon presentation, and the patient denied any blunt trauma to her abdomen. There were no identifiable masses or obstructive stones on the imaging studies. To date, there is no known reason for the patient’s urethral injury, making this a case of truly spontaneous rupture. Any conclusion based upon available studies and history would be purely speculative [7-9].

There are other concerning critical life threats to consider based upon how this patient presented such as aortic dissection and aortic rupture to maintain as well. Additionally, the process should avoid being confused with duodenal or gastric ulcer or perforation which would be more common. The ED treatment includes management of the pain and the associated nausea and emesis. The untreated urinoma can result in complications ranging from sepsis to nephrocutaneous fistulas [2-3].

## Conclusions

The critical part of emergency management for the patient is the identification of the process. In severe cases definitive treatment may be surgical intervention with stent placement and possibly drain placement; in less severe cases observation may be necessary. This is determined by the urology service. Therefore, the emergency physician should keep this in their differential even in the absence of obstructive process, traumatic injuries, or surgical intervention. 
